# Leicester and County Hospital Saturday Society

**Published:** 1917-02-10

**Authors:** 


### Leicester and County Hospital Saturday Society.

### CONTINUOUS PROGRESS AND GOOD BUSINESS.

A striking example of the maxim "Take care of the pence" is to be found in the record of the Leicester and County Hospital Saturday Society for the past year. It is evident from the figures that a period of unexampled commercial prosperity has been enjoyed, for notwithstanding the fact that a goodly number of Territorial regiments have been recruited during the war, the past year's returns exceed those of the previous year by several hundred pounds, and total the large sum of £16,900. The great interest taken by the workpeople in this Fund is seen in the remarkable growth of income during the years for which we give the figures:

| | | £ | | | £ |
|---|---|---|---|---|---|
| 1902 | ... | 4,086 | 1909 | ... | 13,013 |
| 1903 | ... | 5,129 | 1910 | ... | 13,791 |
| 1904 | ... | 7,908 | 1911 | ... | 14,849 |
| 1905 | ... | 9,869 | 1912 | ... | 15,134 |
| 1906 | ... | 11,007 | 1913 | ... | 15,633 |
| 1907 | ... | 11,761 | 1914 | ... | 15,827 |
| 1908 | ... | 12,750 | 1915 | ... | 16,449 |

The expenses of raising this sum of approximately £17,000 are creditably low, being a little under 4 per cent. of the total income. Of the net income of £16,250 seven-tenths is appropriated to the support of the Leicester Royal Infirmary, and the balance to the maintenance of the Society's convalescent homes and other purposes for the welfare of the members. The organising secretary is Mr. H. H. Woolley, who is to be complimented on the manifest success of his organisation.

